# Acetabular labrum blood flow in developmental dysplasia of the hip: an intraoperative in vivo study using laser Doppler flowmetry

**DOI:** 10.1186/s13018-016-0450-6

**Published:** 2016-10-17

**Authors:** So Minokawa, Masatoshi Naito, Koichi Kinoshita, Takuaki Yamamoto

**Affiliations:** Department of Orthopaedic Surgery, Fukuoka University Faculty of Medicine, 7-45-1 Nanakuma, Jonan-ku, Fukuoka 810-0180 Japan

**Keywords:** Developmental dysplasia of the hip (DDH), Periacetabular osteotomy, Acetabular labrum, Blood flow, Laser Doppler flowmetry (LDF)

## Abstract

**Background:**

The vascular supply to the acetabular labrum is important in the treatment of labral lesions. However, in vivo blood flow measurements in the acetabular labrum have not been described in the literature. The purpose of this study was to examine this blood flow in vivo using laser Doppler flowmetry (LDF) in patients with acetabular dysplasia.

**Methods:**

Periacetabular osteotomy combined with arthroscopy was performed in 47 consecutive patients (three males, 44 females; mean age at surgery, 35.6 years; range, 15–60 years). In all patients, blood flow in the acetabular labrum was measured with LDF during arthroscopy. The acetabular labral lesions were categorized according to the modified Beck classification: detachment and full-thickness labral tears were assigned to the T group and normal labrum to the N group. Blood flow rates in the acetabular labrum were compared between the T and N groups. The associations between labral blood flow and the lateral center-edge angle (CEa) and patient age were also evaluated.

**Results:**

The T and N groups comprised 31 and 16 patients, respectively. The mean blood flow rate was 1.94 ± 0.41 ml/min/100 g in the T group and 1.94 ± 0.34 ml/min/100 g in the N group, with no significant difference between the groups (*P* = 0.884). No association was noted between blood flow and either the CEa or patient age (*β* = −0.018, *P* = 0.077 and *β* = −0.001, *P* = 0.770, respectively).

**Conclusions:**

On LDF, blood flow in the acetabular labrum was present in all patients, regardless of the severity of acetabular labral tears, CEa, or age.

## Background

The acetabular labrum, a fibrocartilaginous structure attached to the periphery of the osseous acetabular rim [1], plays an important role in stabilizing the hip joint and protecting the articular cartilage of the hip [2, 3]. The labrum serves as a joint seal that allows joint lubrication and cartilage nutrition, assists in load sharing, and probably plays a role in joint proprioception [2–5]. Developmental dysplasia of the hip (DDH) is known to be associated with intra-articular lesions [6, 7]. Because of the abnormal load-bearing force on the labrum in patients with acetabular dysplasia, the labrum can undergo degeneration, hypertrophy, and tearing [6, 7].

In patients with acetabular dysplasia, arthroscopic labral debridement may fail to provide long-term symptomatic relief and functional improvement because the underlying morphologic hip mechanics remain unchanged [8, 9]. Periacetabular osteotomy was developed to correct the pathologic mechanical environment in the hip [10, 11]; the reported success rate of periacetabular osteotomy in preventing progression of osteoarthritis is approximately 95 % at 5 years after the operation [12]. However, the outcomes of labral lesions after periacetabular osteotomy have not been well documented.

The vascular supply to the labrum is important when considering the optimal treatment for labral lesions. In their study of fresh cadaver hips, Kalhor et al. [13] reported that the blood supply to the acetabular labrum is likely to remain intact in most hips with labral tears. However, in vivo blood flow measurements have not been performed. The purpose of this study was to determine the blood flow in the acetabular labrum in vivo using laser Doppler flowmetry (LDF) in patients with acetabular dysplasia.

## Methods

From March 2014 to December 2015, periacetabular osteotomy combined with arthroscopy was performed on 47 consecutive patients with DDH. The surgical indications for periacetabular osteotomy were dysplastic hips with a lateral center-edge angle (CEa) of <20° in patients with hip pain that interfered with daily activities. The patients comprised three males and 44 females, with a mean age at surgery of 35.6 years (range, 15–60 years). The mean CEa, as measured on preoperative plain radiographs, was 12.0° (range, −2.9° to 20.0°). The stage of osteoarthritis was graded radiologically according to the Tönnis classification [14]: 38 hips were grade 0 and nine hips were grade 1. Acetabular labral tears were categorized according to Beck’s modified classification [15]: detachment and full-thickness labral tears were assigned to the T group and normal labrum to the N group. The locations of the labral lesions were categorized into three regions [16]: anterosuperior (AS), superior (SU), and posterosuperior (PS). No patients had vasculopathy, and none were administered anticoagulants before surgery.

The operations were performed by a single hip surgeon (MN) at our facility. The blood flow in the acetabular labrum in the 47 patients was measured with LDF (ALF21N; ADVANCE Co., Tokyo, Japan) during arthroscopy. The same investigator (SM) took the blood flow measurements in all cases. LDF is used to noninvasively and repeatedly measure tissue blood flow. This technique has proven useful for clinical and experimental evaluation of blood flow in the skin, ligaments, anterior and posterior lesions of the superior labrum, and bone [17, 18].

**Fig. 1:**
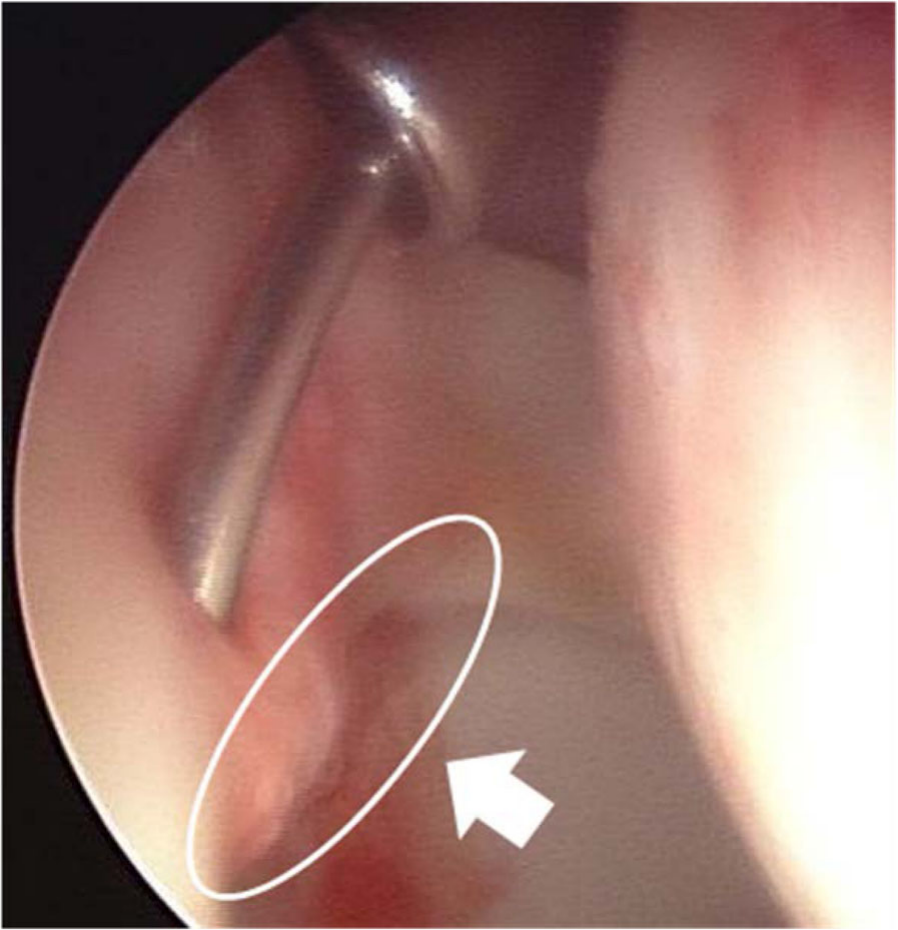
All of the detached labral lesions were located in the anterosuperior area (*white arrow* and *circle*). The LDF probe tip was placed on the detached labral lesions

Surgery was performed with the patient under general anesthesia and lying on a traction table. The hip joint structure was arthroscopically observed through an anterolateral portal, and the hook-probe and LDF probe were inserted through a mid-anterior portal. Arthroscopic surgery was performed with the aid of a light source (LS7700; ConMed Linvatec Corporation, Largo, FL). During arthroscopic surgery, fluid management was achieved with air only under negative pressure; a perfusate system was not used. We evaluated the condition of the labrum (normal, detached, or full-thickness tear) without performing labrum resection or repair. After evaluation of the condition of the labrum, the LDF probe was placed in contact with the torn or normal acetabular labrum, and blood flow was measured within a 2-mm hemisphere from the center of the probe tip (Fig. 1). The LDF probe was placed on the same labral area (AS area) throughout measurement in all patients. Blood flow was measured at the surface 3 to 5 mm from the acetabular articular cartilage of normal labrum and in detached labral lesions. For the three patients with full-thickness bucket-handle tears, blood flow was measured at the base on the acetabular rim side. Blood pressure was maintained at a normal level (systolic blood pressure <130 mmHg), as established by the World Health Organization and International Society of Hypertension. We recorded the systolic blood pressure when the LDF probe was placed in contact with the acetabular labrum. Blood flow measurements were repeated three times, and the mean values were calculated.

We compared blood flow rates in the acetabular labrum between the T and N groups. Additionally, we used multiple regression analysis to examine the effects of CEa and patient age on blood flow, with CEa, patient age, labral type (N or T group), and systolic blood pressure as independent variables. Data are presented as the mean ± standard deviation. Statistical analyses were performed with SPSS software, version 20 (SPSS Inc., Chicago, IL). The Mann–Whitney *U* test was used to compare mean values between the two groups. Values of *P* < 0.05 were considered statistically significant.

## Results

The T group comprised 31 patients (three males, 28 females; detachment, *n* = 28; full-thickness tear, *n* = 3) and the N group comprised 16 patients (0 males, 16 females). The labral tears were most commonly located in the AS area (100 %, 31/31 hips), followed by the SU area (51.6 %, 16/31 hips), and the PS area (16.1 %, 5/31 hips). Detached lesions were located in the AS area (100 %, 28/28 hips), SU area (35.7 %, 10/28 hips), and PS area (17.9 %, 5/28 hips). Full-thickness tears were located in the AS area (100 %, 3/3 hips) and SU area (33.3 %, 1/3 hips). The LDF probe was placed in the same labral area (AS area) throughout measurement in all patients.

The mean age at the time of surgery was 38.8 years in the T group and 29.6 years in the N group (*P* = 0.039). The mean preoperative CEa was 13.6° in the T group and 11.2° in the N group (*P* = 0.50). Mean systolic blood pressure was 85.9 ± 5.3 mmHg in the T group and 86.0 ± 6.9 mmHg in the N group (*P* = 0.50). The mean blood flow rate was 1.94 ± 0.41 ml/min/100 g in the T group and 1.94 ± 0.34 ml/min/100 g in the N group (*P* = 0.884) (Table 1). No association was noted between labral blood flow and either CEa or patient age (*β* = −0.018, *P* = 0.077 and *β* = −0.001, *P* = 0.770, respectively) (Table 2).

**Table 1:** Patient demographic data and blood flow rates according to group

Parameter	N group	T group	*P* value
No. of hips	16	31	–
Gender (male/female)	All female	3:28	–
Age (range) (years)	29.6 ± 9.4	38.8 ± 13.7	0.039
CE angle (°)	13.6 ± 5.3	11.2 ± 5.9	0.500
Systolic blood pressure (mmHg)	85.9 ± 5.3	86.0 ± 6.9	0.946
Blood flow data (ml/min/100 g)	1.94 ± 0.34	1.94 ± 0.41	0.884

Data are presented as mean ± standard deviation unless otherwise indicated

*CE* center-edge

**Table 2:** Results of multiple regression analysis

	Blood flow	
	*β*	*P* value
CE angle	−0.018	0.077
Age	−0.001	0.77
Systolic blood pressure	0.02	0.027
Labral type (N/T group)	−0.042	0.73

*CE*, center-edge, *N/T group* normal/tear group, *β* regression coefficient

## Discussion

A fresh cadaveric study showed that the acetabular labrum is supplied by radial branches of a periacetabular vascular ring; regardless of the depth and extent of labral lesions, the capsular side of the labrum remains in continuity with the acetabular rim and the overlying vascular connective tissue layer remains intact [15]. The results of our study support the findings of that cadaveric study, suggesting that the blood supply to the acetabular labrum remains intact in most hips with labral tears [15].

In this study, blood flow to the labrum did not correlate with either CEa or patient age. Furthermore, blood flow in torn labrum was approximately equal to that in normal labrum. If blood flow to the labrum decreases, the free edge is likely to become ischemic. This change can lead to tissue vulnerability, which could result in degeneration and fraying of the labrum. However, we found that blood flow in the acetabular labrum was present in all patients, regardless of the degree of DDH, age, or severity of acetabular labral tears. These findings suggest that a torn acetabular labrum in patients with DDH has the capacity to heal.

Treatment of labral tears in patients with acetabular dysplasia remains controversial. Byrd and Jones [19] reported that short-term functional outcome scores were similar in patients undergoing combined arthroscopic labral repair and periacetabular osteotomy versus periacetabular osteotomy alone. However, postoperative improvements in patient-reported outcomes were seen in the combined arthroscopic labral repair and periacetabular osteotomy group, and this group had greater improvement in quality-of-life scores at latest follow-up as compared with patients who underwent periacetabular osteotomy alone [19]. Therefore, our findings may have implications for the surgical repair of labral tears in DDH patients undergoing periacetabular osteotomy, because we found that blood flow was present in the acetabular labrum in all patients.

DDH is a common cause of secondary osteoarthritis [20]. In the presence of a shallow bony acetabulum, the labrum may become hypertrophic, assuming a more important role as a weight-bearing surface and taking on added responsibility for joint stability. These abnormalities can lead to subsequent labral tears and degeneration of the articular cartilage [4, 18]. Dorrell and Catterall [8] reported that arthroscopic labral debridement can fail to provide long-term symptomatic relief and functional improvement if the underlying abnormal hip mechanics in DDH are not addressed. This is supported by findings from Kain et al. [21], who found that periacetabular osteotomy performed to restore acetabular coverage provided good functional results in patients who had failed to improve after undergoing isolated arthroscopic labral debridement for hip symptoms in the setting of acetabular dysplasia.

Our study has three main limitations. First, it included a small number of patients, all of whom had DDH. The hip joint conditions were not normal, which may have affected blood flow measurements. Additionally, in our study, the blood flow was evaluated only in patients with acetabular dysplasia. It is unclear whether our findings are relevant in the treatment of labral tears in patients without dysplasia, including those with femoroacetabular impingement. Second, our study lacked follow-up data on postoperative clinical outcomes. However, the focus of this study was the evaluation of blood flow in the labrum. Third, blood flow was measured only in the anterosuperior portion because it was technically difficult to cover the entire acetabular labrum lesion with the LDF probe.

## Conclusions

In conclusion, the results of our study support the findings of a prior study suggesting that the blood supply to the acetabular labrum remains intact in most hips with labral tears. Regardless of the degree of DDH, age, and severity of acetabular labral tears, blood flow in the acetabular labrum was present in all patients. Blood flow rates in the acetabular labrum may have implications for the healing capacity of labral tears after periacetabular osteotomy in patients with DDH. Combined surgical repair of labral tears seems to be the useful option for the patients undergoing periacetabular osteotomy.

## Abbreviations

CEa: Lateral center-edge angle
DDH: Developmental dysplasia of the hip
LDF: Laser Doppler flowmetry
